# Silicon–silicon π single bond

**DOI:** 10.1038/s41467-020-17815-z

**Published:** 2020-08-11

**Authors:** Soichiro Kyushin, Yoshikuni Kurosaki, Kyohei Otsuka, Haruna Imai, Shintaro Ishida, Toru Kyomen, Minoru Hanaya, Hideyuki Matsumoto

**Affiliations:** 1 Division of Molecular Science, Graduate School of Science and Technology, Gunma University, Kiryu, Gunma 376-8515 Japan; 2 Department of Chemistry, Graduate School of Science, Tohoku University, Sendai, 980-8578 Japan; 3 Division of Pure and Applied Science, Graduate School of Science and Technology, Gunma University, Maebashi, Gunma 371-0044 Japan

**Keywords:** Chemical bonding, Stereochemistry

## Abstract

A carbon–carbon double bond consists of a σ bond and a π bond. Recently, the concept of a π single bond, where a π bond is not accompanied by a σ bond, has been proposed in diradicals containing carbon and heteroatom radical centers. Here we report a closed-shell compound having a silicon–silicon π single bond. 1,2,2,3,4,4-Hexa-*tert*-butylbicyclo[1.1.0]tetrasilane has a silicon−silicon π single bond between the bridgehead silicon atoms. The X-ray crystallographic analysis shows that the silicon−silicon π single bond (2.853(1) Å) is far longer than the longest silicon−silicon bond so far reported. In spite of this unusually long bond length, the electrons of the 3p orbitals are paired, which is confirmed by measurement of electron paramagnetic resonance, and magnetic susceptibility and natural bond orbital analysis. The properties of the silicon−silicon π single bond are studied by UV/Vis and ^29^Si NMR spectroscopy, and theoretical calculations.

## Abstract (web summary)

Examples of π single bonds in diradicals are rare, as they are typically unstable. Here, the authors describe a room temperature-stable closed-shell bicyclic tetrasilane that features a Si-Si π single bond between two of the non-adjacent Si centers.

## Introduction

**Fig. 1:**
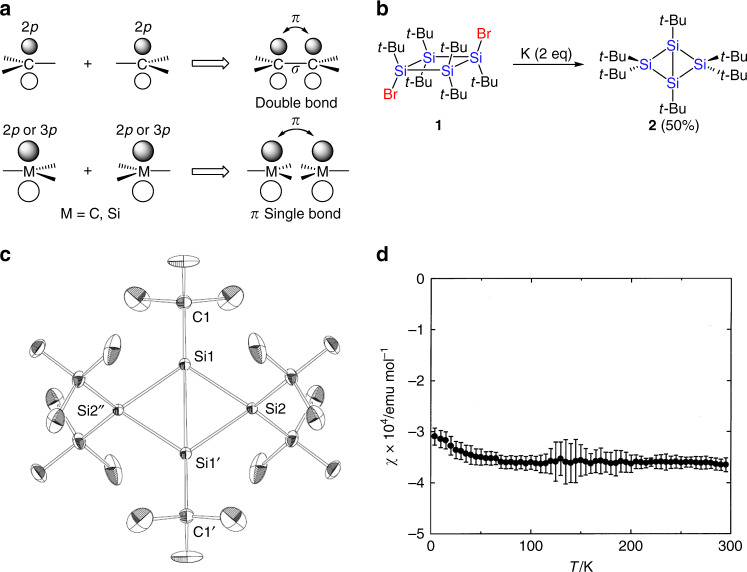
Synthesis, structure, and magnetic property of 2. **a** Concepts of a double bond (top) and a π single bond (bottom). **b** Synthesis of **2**. **c** Molecular structure of **2**. Thermal ellipsoids are drawn at the 30% probability level. Selected bond length (Å) and angle (°): Si1–Si2 2.3090(7), Si1–Si1’ 2.853(1), Si1–C1 1.921(3); Si2–Si1–Si1’ 51.83(2), Si2–Si1–Si2” 103.65(4), Si2–Si1–C1 128.07(2), Si1’–Si1–C1 176.1(1), Si1–Si2–Si1’ 76.35(4). **d** Temperature-dependent magnetic susceptibility of **2**. The data were measured 12 times at each temperature. The black dots denote average values and the error bars show SDs.

The concept of a carbon–carbon double bond is one of the fundamentals in organic chemistry. A carbon–carbon double bond is formed by overlapping of the *sp*^2^ orbitals (σ bond) and 2*p* orbitals (π bond). In this double bonding, two *sp*^2^ carbon atoms are faced in the direction of the *sp*^2^ orbital. Another possible bond is a π single bond, where two *sp*^2^ carbon atoms are faced in the bisecting direction between two *sp*^2^ orbitals (Fig. 1a). This new π single bond has recently been reported to present in singlet diradicals with π single bond character and stabilization of such singlet diradicals have been challenged^1–3^. On the other hand, studies on singlet diradicals consisting of heteroatoms have recently been developed^4–6^. For example, the singlet diradical [B(*t*-Bu)]_2_[P(*i-*Pr)_2_]_2_ was isolated as a stable singlet diradical and its structure and properties have been reported^7^.

We report herein that 1,2,2,3,4,4-hexa-*tert*-butylbicyclo[1.1.0]-tetrasilane having a silicon–silicon π single bond was isolated as a closed-shell compound (Nukazawa and Iwamoto^8^ have quite recently reported bicyclo[1.1.0]tetrasilane with a planar structure, and Scheschkewitz and colleagues have quite recently reported 1,3-cyclotetrasilanediyl having a π single bond^9^). The structural features and electronic properties of the silicon–silicon π single bond were revealed by X-ray crystallography, electron paramagnetic resonance (EPR), magnetic susceptibility, ultraviolet/visible (UV/Vis), ^29^Si nuclear magnetic resonance (NMR), and theoretical calculations. On the basis of these results, we propose this bond as a member of silicon–silicon bonds such as single bonds, double bonds^10–17^, and triple bonds^18–22^.

## Results

### Synthesis, structure, and properties

The reduction of *trans*-1,3-dibromo-1,2,2,3,4,4-hexa-*tert*-butylcyclotetrasilane (**1**) with a large excess of potassium has previously been reported to give the corresponding 1,3-dipotassiocyclotetrasilane^23^. When the reduction of **1** was carried out with two equivalents of potassium, 1,2,2,3,4,4-hexa-*tert*-butylbicyclo[1.1.0]tetrasilane (**2**) was obtained as orange crystals in 50% yield (Fig. 1b). Compound **2** is stable at room temperature under an argon atmosphere in a glovebox, but it is decomposed in air.

The structure of **2** was determined by X-ray crystallography (Fig. 1c and Supplementary Fig. 7). This molecule has the *C*_2*h*_ symmetry and, therefore, the bicyclo[1.1.0]tetrasilane moiety has a completely planar structure with the interflap angle between the two cyclotrisilane rings being 180.0°. The distance between the bridgehead silicon atoms is 2.853(1) Å. This value is far larger than the longest silicon–silicon σ bonds (2.697 Å^24^ and 2.7288(15) Å^25^) and distances between inverted tetrahedral silicon atoms (2.636(1) Å^26^ and 2.7076(8) Å^27^). It is noted that the silicon–silicon and germanium–germanium double bonds in bicyclo[1.1.0]tetrasil-1(3)-ene and a related Ge_2_Ga_2_ compound have a π bond and a weak σ bond, and are considerably elongated^28,29^. The Si(1)–Si(2) bond length (2.3090(7) Å) is shorter than normal silicon–silicon bonds (2.358 Å)^30^, probably due to the connection of the two bridgehead silicon atoms. The bridgehead silicon atoms have an almost planar structure: the sum of Si2–Si1–Si2” (103.65(4)°), Si2−Si1−C1 (128.07(2)°), and Si2”–Si1–C1 (128.07(2)°) bond angles is 359.79°. The planar structure of the bridgehead silicon atoms shows that these silicon atoms are nearly *sp*^2^-hybridized, which is also discussed later by natural bond orbital (NBO) analysis.

A question comes from this planar structure: can the electron in the 3*p* orbital of the bridgehead silicon atom interact with each other in spite of this unusually long distance? To clarify this point, we measured EPR spectra and magnetic susceptibility. Compound **2** showed no EPR signals in solid state and in a benzene solution (Supplementary Figs. 5 and 6). The values of the magnetic susceptibility are negative in the temperature range from 4 to 300 K (Fig. 1d). As no temperature dependence was observed, the magnetic susceptibility does not obey the Curie law. These results clearly show that **2** is not a paramagnetic compound such as a triplet silyl biradical but a diamagnetic compound with a singlet state.

**Fig. 2:**
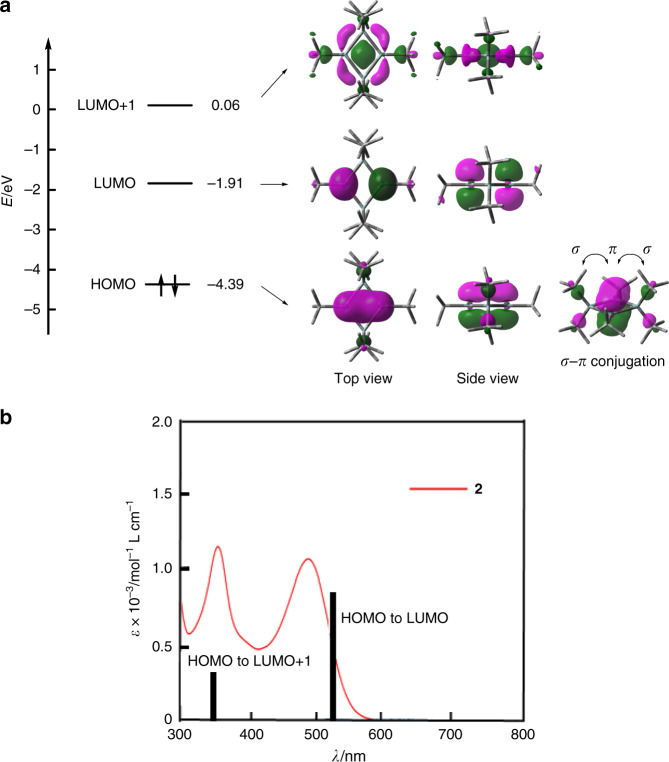
Electronic properties of 2. **a** Frontier orbitals and their energy levels of **2** calculated at the B3LYP/6-31G(d) level. **b** UV/Vis spectrum of **2** in benzene at room temperature with transitions calculated at the TD-DFT B3LYP/6-31G(d) level.

The electronic interaction between the bridgehead silicon atoms was studied by theoretical calculations. The optimized structure of **2** well reproduces the planar structure (Supplementary Tables 1 and 2). The highest occupied molecular orbital (HOMO) and the lowest unoccupied molecular orbital (LUMO) are shown in Fig. 2a. In the HOMO, the 3*p* orbitals of the bridgehead silicon atoms overlap to form a π bond. Four silicon–carbon σ orbitals contribute to the HOMO, showing the σ–π conjugation between the π–orbital and the four silicon–carbon σ orbitals. The LUMO is a π* orbital with a nodal plane. The NBO analysis was also carried out (Supplementary Data 1–3). The calculation showed that the hybrid orbital of the bridgehead silicon atom used for the bonds to the neighboring silicon and carbon atoms has 33.57% (Si) and 32.42% (C) of *s*-characters and 66.06% (Si) and 66.87% (C) of *p*-character, indicating *sp*^2^ hybrid orbitals. The orbital used for the silicon–silicon π single bond has 0.32% of *s*-character and 99.46% of *p*-character. Wiberg bond index of the silicon–silicon π single bond is 0.67. These results show that **2** is a compound with a silicon–silicon π–single bond rather than a singlet diradical.

We obtained other experimental evidence for the silicon–silicon π single bond. The UV/Vis spectrum of **2** is shown in Fig. 2b and Supplementary Fig. 4. Compound **2** shows absorption maxima at 351 and 485 nm. These absorption maxima are assigned to the HOMO to LUMO (485 nm) and HOMO to LUMO + 1 (351 nm) transitions on the basis of a time-dependent density functional theory (TD-DFT) calculation (Supplementary Data 4). The lowest energy absorption maximum at 485 nm is in the similar wavelength region compared with planar disilenes such as [(*i*-Pr)_2_MeSi]_2_Si = Si[SiMe(*i*-Pr)_2_]_2_ (*λ*_max_ 412 nm)^31^, [(*t*-Bu)Me_2_Si]_2_Si = Si[SiMe_2_(*t*-Bu)]_2_ (*λ*_max_ 424 nm)^31^, [(*i*-Pr)_3_Si]_2_Si = Si[Si(*i*-Pr)_3_]_2_ (*λ*_max_ 484 nm)^31^, and bicyclo[3.3.0]octasil-1(5)-ene (*λ*_max_ 468 nm)^32^. Probably, the presence or absence of the silicon–silicon σ bond does not affect the π–π* transition considerably.

In the ^29^Si NMR spectrum, **2** shows the signal of the bridgehead silicon atoms at *δ* 117.4 ppm (Supplementary Fig. 3). In the case of disilenes, the signals of *sp*^2^ silicon atoms of most tetraalkyl- and tetraaryldisilenes have been reported to be observed at 50–100 ppm, while those of tetrasilyldisilenes show a large downfield shift (*δ* 140–170 ppm)^12,14,16^. The signal of **2** at *δ* 117.4 ppm exists in the intermediate region. This result is explained by substituents on *sp*^2^ silicon atoms. Each *sp*^2^ silicon atom of tetraalkyl- and tetraaryldisilenes is attached by two carbon atoms and one silicon atom, whereas each *sp*^2^ silicon atom of tetrasilyldisilenes is attached by three silicon atoms. In the case of **2**, each *sp*^2^ silicon atom is attached by two silicon atoms and one carbon atom. Therefore, the intermediate *δ* value of **2** might be reasonable.

### Analysis of the planar structure

The planar structure of **2** is interesting from the following viewpoints. Table 1 shows the X-ray structural parameters of **2** and related cyclotetrasilanes **1**, **3**, and **4**^23,33^. These cyclotetrasilanes have been reported to have planar cyclotetrasilane rings and, therefore, the steric repulsion between neighboring *tert*-butyl groups is large due to the eclipsed conformation. When the van der Waals radius of the substituents on the 1,3-silicon atoms (i.e., H, Br, and K) becomes smaller, the geometry of the 1,3-silicon atoms change from pyramidal to trigonal monopyramidal structures as shown by the Σ (Si) values. This is caused by the reduction of the steric repulsion between neighboring *tert*-butyl groups. When the hydrogen atoms on the silicon atoms of **3** are removed, the silicon atoms of **2** become nearly planar. Table 1 also shows the Si–Si–Si bond angles. As the van der Waals radius of the substituents on the 1,3-silicon atoms becomes smaller, the *θ*_1_ value becomes slightly larger and the *θ*_4_ value becomes slightly smaller, because of the Thorpe–Ingold effect of the SiX(*t*-Bu) and Si(*t*-Bu)_2_ moieties^34–36^; finally, the cyclotetrasilane ring becomes rhombohedral in **2**^37^. The increment of the *θ*_1_ value from **3** (93.7(1)°) to **2** (103.65(4)°) is significantly large in this series. This structural change corresponds to the formation of the transannular silicon–silicon π single bond.

**Table 1:** Comparison of X-ray structural parameters of **1**–**4**.


Compound	2	3^33^	1^23^	4^23^
van der Waals radius of *X*/Å^38^	–	1.20	1.85	2.75
Σ (Si)/°	359.79	350.3	345.9	334.5
*θ*_1_/°	103.65(4)	93.7(1)	93.17(3)	90.68(5)
*θ*_2_/°	128.07(2)	128.3(1)	126.4(1)	121.9(2)
*θ*_3_/°	128.07(2)	128.3(1)	126.3(1)	121.9(2)
*θ*_4_/°	76.35(4)	86.4(1)	86.83(3)	89.44(8)

The structure of **2** is also interesting from the viewpoint of the bond-stretch isomers of bicyclo[1.1.0]tetrasilane. It was predicted by theoretical calculations that bicyclo[1.1.0]tetrasilane has two bond-stretch isomers, i.e., a short-bond isomer and a long-bond isomer^38–45^. The short-bond isomer has a small bridgehead Si–Si bond length (*r*), a small fold angle between two three-membered rings (*ϕ*), and a large H−Si−Si bond angle (*θ*). On the other hand, the long-bond isomer has large *r*, large *ϕ*, and small *θ* values (Supplementary Tables 1 and 2). In fact, short-bond isomers of bicyclo[1.1.0]tetrasilanes^46,47^ and a long-bond isomer of 1,3-disilabicyclo[1.1.0]butane^48^ have been reported. The structural features of **2** are quite different from these bond-stretch isomers. To understand why **2** becomes a different bond-stretch isomer, we studied substituent effects by theoretical calculations.

**Fig. 3:**
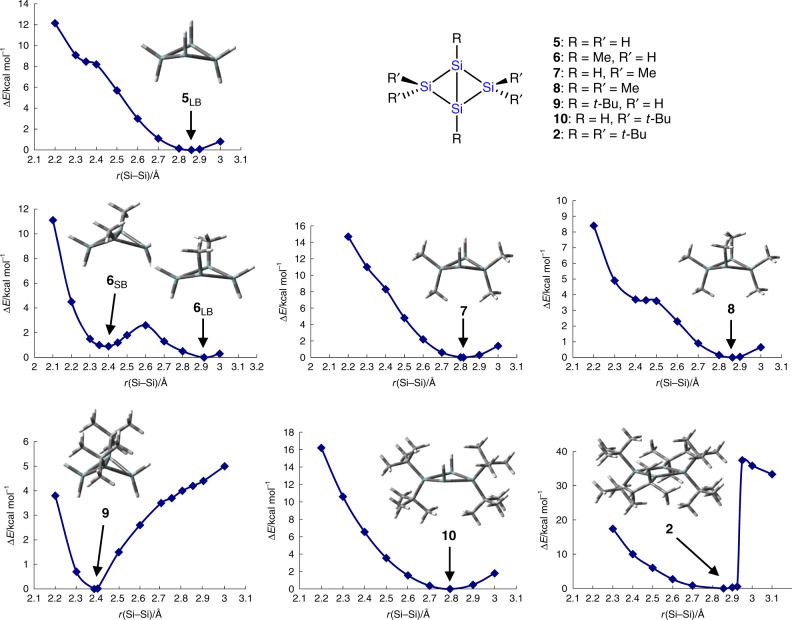
Energy curves of 2 and 5–10 calculated at the B3LYP/6-31G(d) level. The terms SB and LB denote short-bond and long-bond isomers, respectively.

Figure 3 shows the energies of optimized structures of bicyclo-[1.1.0]tetrasilane (**5**) at various bridgehead silicon–silicon bond lengths. The structures at the inflection point at 2.35 Å and the energy minimum at 2.86 Å correspond to the short- and long-bond isomers (Supplementary Tables 1 and 2). When methyl groups are introduced, the energy curve is greatly affected. The long-bond isomer of **6** is considerably destabilized because of the steric repulsion between the bridgehead methyl groups. On the other hand, the short-bond isomer of **7** is destabilized due to the steric hindrance between the faced *tert*-butyl groups on the folded two cyclotrisilane rings. In the case of **8**, both destabilization effects work, and as a result, the energy curve resembles that of **5** to some extent.

We also calculated the energy curves of **9**, **10**, and **2** with *tert*-butyl groups (Fig. 3 and Supplementary Tables 1 and 2). The energy curves of **9** and **10** show more pronounced features of **6** and **7**: the long-bond isomer of **9** is more destabilized than its short-bond isomer and the short-bond isomer of **10** is more destabilized than its long-bond isomer. This is due to the large steric effect of the *tert*-butyl group. When six *tert*-butyl groups are attached (**2**), both short-bond and long-bond isomers are highly destabilized. Instead, the planar structure becomes the energy minimum. In the planar structure, the steric repulsion among the bridgehead *tert*-butyl groups and those at the 2,2,4,4-positions can be avoided. Therefore, the planar isomer may be added to the bond-stretch isomers as a different isomer.

In conclusion, bicyclo[1.1.0]tetrasilane having a silicon–silicon π single bond was synthesized by introduction of six *tert*-butyl groups. As this compound was isolated as stable crystals, synthesis of related compounds may be possible. Such studies are now in progress.

## Methods

### 1,2,2,3,4,4-Hexa-*tert*-butylbicyclo[1.1.0]tetrasilane (2)

A mixture of **1** (41.1 mg, 6.68 × 10^−2^ mmol), potassium (5.5 mg, 0.14 mmol), benzene (6 mL), and tetrahydrofuran (THF) (4 mL) was stirred at 50 °C for 2 h in a glovebox. Insoluble materials were removed by filtration through a glass filter. The filtrate was concentrated by slow evaporation of the solvents to give **2** (15.1 mg, 50%) as orange crystals. **2**. ^1^H NMR (500 MHz, toluene-*d*_8_): *δ* 1.30 (s, 36H), 1.32 (s, 18H). ^13^C NMR (126 MHz, toluene-*d*_8_): *δ* 24.7, 28.5, 32.3, 33.9. ^29^Si NMR (99 MHz, toluene-*d*_8_): *δ* 13.7, 117.4. UV/Vis (benzene): *λ*_max_ (*ε*) 351 (1110 mol^−^^1^ L cm^−1^), 485 nm (1040). High-resolution mass spectrometry: found 454.3307, calcd for C_24_H_54_Si_4_ 454.3303.

### X-ray crystallographic analysis

Orange crystals of **2** were obtained from a benzene–THF (6 : 4) solution by slow evaporation under an argon atmosphere in a glovebox. A crystal specimen was mounted in a loop and was used for data collection on a Rigaku R-AXIS IV^++^ imaging plate diffractometer using graphite-monochromated Mo Kα radiation. The data were corrected for Lorentz and polarization effects. An empirical absorption correction based on multi-scan was also applied. The structure was solved by a direct method using SHELXS-97^49^. Non-hydrogen atoms were refined anisotropically by the full-matrix least-squares method on *F*^2^ for all reflections using SHELXL-2014/7^49,50^. All hydrogen atoms were generated by AFIX instructions and were not refined. All calculations were carried out using Yadokari-XG 2009^51^. Crystal data for **2** (123 K): C_24_H_54_Si_4_, fw = 455.03, monoclinic, space group *C*2/*m*, *a* = 16.662(3), *b* = 11.5749(17), *c* = 8.5032(15) Å, *β* = 115.5534(9)°, *V* = 1479.5(4) Å^3^, *Z* = 2, *D*_calcd_ = 1.021 g cm^−3^, *R*1 = 0.053 (all data), *wR*_2_ = 0.129 (all data), goodness-of-fit = 1.11.

### Theoretical calculations

All theoretical calculations were performed using Gaussian 09^52^ on a Fujitsu PRIMERGY RX300 system of the Research Center for Computational Science, Japan. The structures were optimized at the B3LYP/6-31G(d) level and the optimization was confirmed by frequency calculations. The results are summarized in Supplementary Tables 1 and 2. The NBO analysis of **2** was carried out at the B3LYP/6-31 + G(d,p) level using the X-ray structure. The result is summarized in Supplementary Data 1–3. The TD-DFT calculation of **2** was performed at the B3LYP/6-31G(d) level using the optimized structure. The result is summarized in Supplementary Data 4.

## Supplementary information

Supplementary Information

Peer Review File

Description of Additional Supplementary Information

Supplementary Data 1

Supplementary Data 2

Supplementary Data 3

Supplementary Data 4

## Data Availability

The data supporting this study are available within this paper, Supplementary Information, and Supplementary Data. CCDC 1997577 (**2**) contains the supplementary crystallographic data for this paper. These data can be obtained free of charge from The Cambridge Crystallographic Data Centre.
