# Supplementary material for: Silicon–silicon π single bond
Source: Nat Commun. 2020 Aug 11;11:4009. doi: 10.1038/s41467-020-17815-z (PMC7419521; doi:10.1038/s41467-020-17815-z)
Supplement: Supplementary file 1 — Supplementary Information [file 41467_2020_17815_MOESM1_ESM.pdf]

Supplementary Information

Silicon–silicon π single bond

Kyushin et al.

Supplementary Methods

1. Experimental Details

Reaction. All experiments were performed under an argon atmosphere. Benzene was dried over sodium, distilled from lithium aluminum hydride, and stored over a potassium mirror. THF was dried over sodium, distilled from sodium benzophenone ketyl, and stored over a potassium mirror. Potassium (Aldrich) was purchased and used without further purification.

Measurements. ^{1}H (500 MHz), ^{13}C (126 MHz), and ^{29}Si (99 MHz) NMR spectra were measured with a JEOL JNM-LA500 spectrometer. EPR spectra were obtained with a JEOL JES-RE2X spectrometer. Magnetic susceptibility was measured on a Quantum Design MPMS5-SW SQUID magnetometer in Industry Collaboration and Intellectual Property Strategy Center, Gunma University. A UV/Vis spectrum was measured on a JASCO V-570 spectrophotometer with a 1 cm quartz cell. A high-resolution mass spectrum was recorded on JEOL JMS-T100GCV mass spectrometer.

Synthesis of *trans*-1,1,2,3,3,4-hexa-*tert*-butylcyclotetrasilane (3).[1]

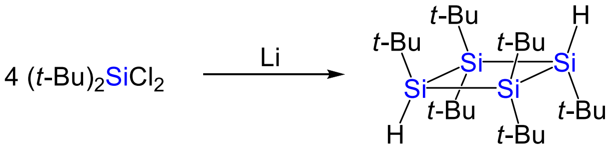

3 (24%)

This compound was synthesized by a modified procedure of reference 1. A mixture of di-*tert*-butyldichlorosilane (24.5 g, 115 mmol) and lithium powder (2.12 g, 305 mmol) in THF (100 mL) was stirred at room temperature for 72 h. A large amount of methanol was added to the reaction mixture at 0 °C. The solvents were removed by evaporation, and the residue was dissolved in hexane and passed through a short column over silica gel. The eluate was evaporated, and the residue was recrystallized from methanol–THF (4:6) to give **3** (3.16 g, 24%) as colorless crystals.

3. Mp: 221–224 °C. ^{1}H NMR (C_6D_6): δ 1.26 (s, 18H), 1.32 (s, 36H), 4.20 (s, 2H). ^{13}C NMR (C_6D_6): δ 21.3, 23.5, 32.3, 33.0. ^{29}Si NMR (C_6D_6): δ –35.1, 32.0. IR (KBr): 2920, 2850, 2060, 1455, 1385, 1360, 1180, 1010, 815, 770, 675 cm^{-1}. MS (EI): 456 (M^{+}, 6), 399 (29), 343 (16), 287 (10), 73 (100). UV (hexane): λ_{max} (ε) 275 nm (1370 mol^{-1} L cm^{-1}).

Synthesis of *trans*-1,3-dibromo-1,2,2,3,4,4-hexa-*tert*-butylcyclotetrasilane (1).[2]

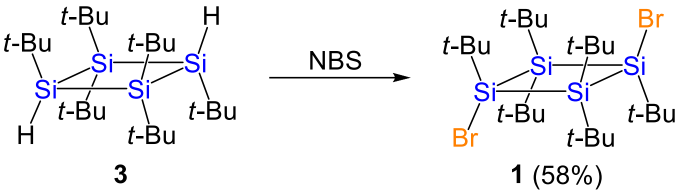

A solution of **3** (2.01 g, 4.39 mmol) and NBS (6.26 g, 35.2 mmol) in THF (200 mL) was heated at 70 °C for 2 days. After the solvent was removed by evaporation, the residue was dissolved in hexane, and the solution was passed through a short column over silica gel. The eluted material was recrystallized from methanol–THF to give **1** (1.55 g, 58%) as colorless crystals.

1. Mp: 182–184 °C. ^{1}H NMR (C_6D_6): δ 1.41 (s, 18H), 1.45 (s, 36H). ^{13}C NMR (C_6D_6): δ 25.8, 26.9, 30.8, 33.4. ^{29}Si NMR (C_6D_6): δ 24.7, 40.7. IR (NaCl): 2940, 2850, 1460, 1390, 1360, 1180, 1010, 810 cm^{-1}. UV (hexane): λ_{max} (ε) 291 (480 mol^{-1} L cm^{-1}), 358 nm (140). MS: *m/z* 612 (M^+($^{79}Br_2$), 17), 73 (73), 57 (100). Anal. Found: C, 46.60; H, 8.69. Calcd for $C_{24}H_{54}Br_2Si_4$: C, 46.88; H, 8.85.

Synthesis of 1,2,2,3,4,4-hexa-*tert*-butylbicyclo[1.1.0]tetrasilane (2).

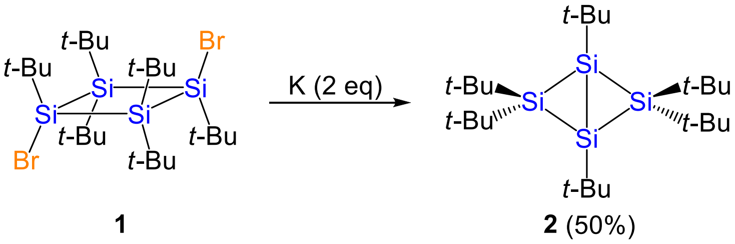

A mixture of **1** (41.1 mg, 6.68 × 10^{-2} mmol), potassium (5.5 mg, 0.14 mmol), benzene (6 mL), and THF (4 mL) was stirred at 50 °C for 2 h in a glovebox. Insoluble materials were removed by filtration through a glass filter. The filtrate was concentrated by slow evaporation of the solvents to give **2** (15.1 mg, 50%) as orange crystals.

2. ^{1}H NMR (500 MHz, toluene-d_8): δ 1.30 (s, 36H), 1.32 (s, 18H). ^{13}C NMR (126 MHz, toluene-d_8): δ 24.7, 28.5, 32.3, 33.9. ^{29}Si NMR (99 MHz, toluene-d_8): δ 13.7, 117.4. UV/Vis (benzene): λ_{max} (ε) 351 (1110 mol^{-1} L cm^{-1}), 485 nm (1040). HRMS: found 454.3307, calcd for $C_{24}H_{54}Si_4$ 454.3303.

2. Spectral Data

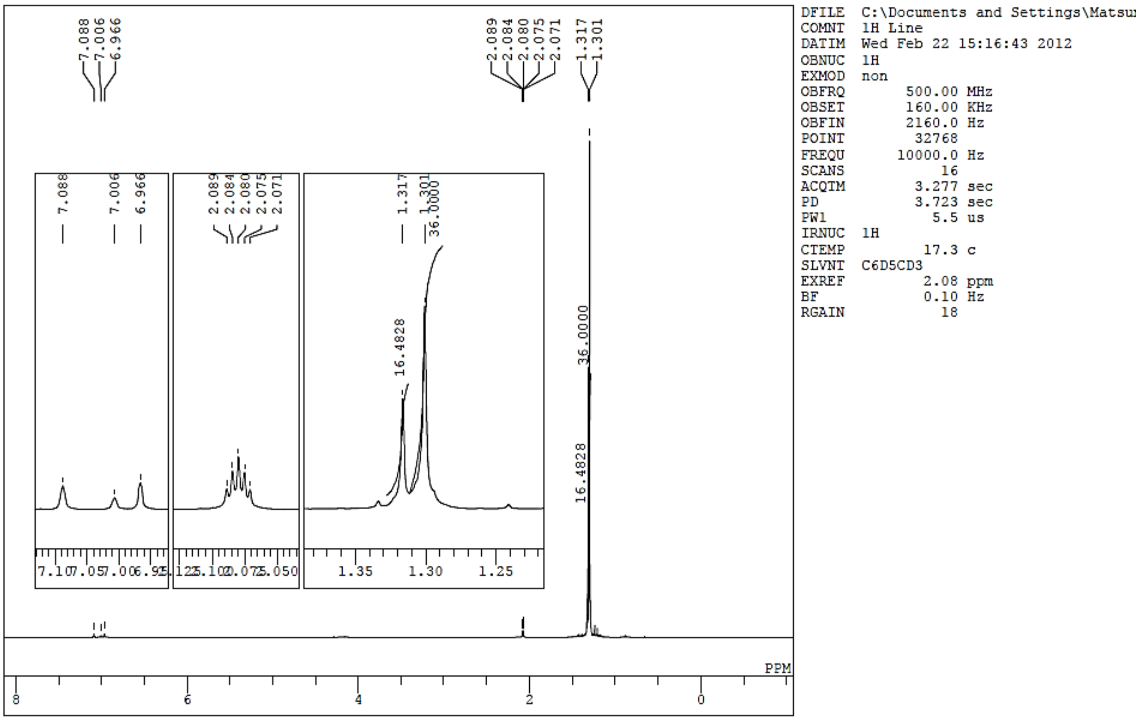

Supplementary Fig. 1 ^{1}H NMR spectrum of 2 in toluene-d_8 at room temperature.

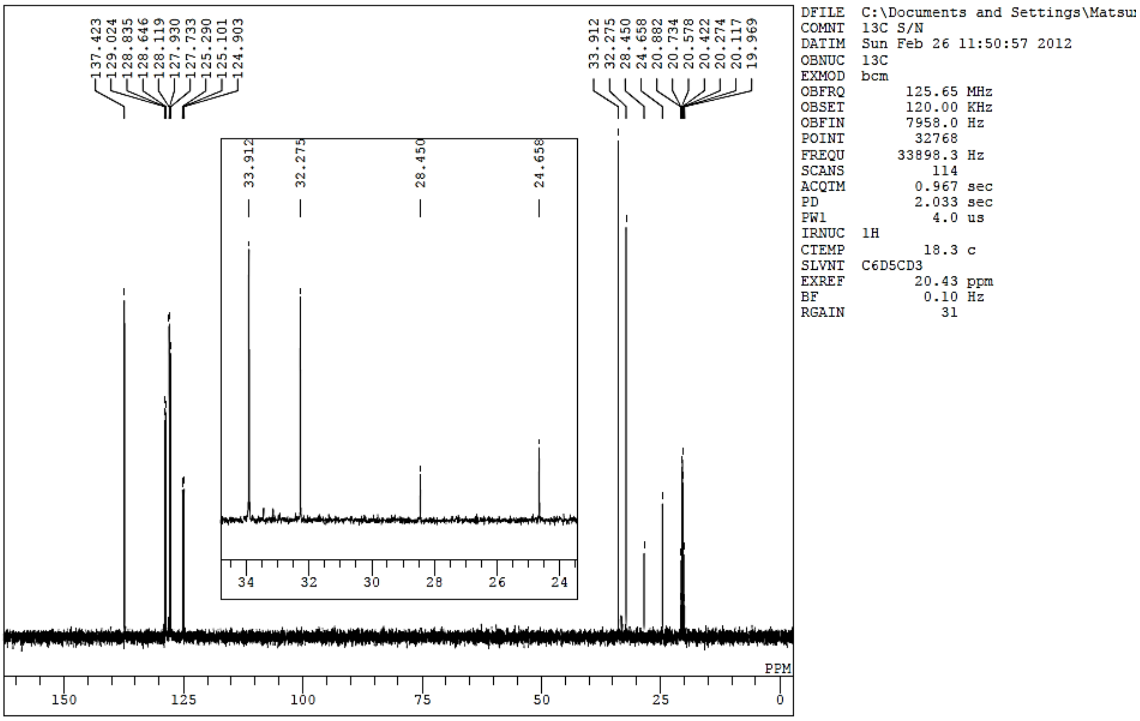

Supplementary Fig. 2 ^{13}C NMR spectrum of 2 in toluene-d_8 at room temperature.

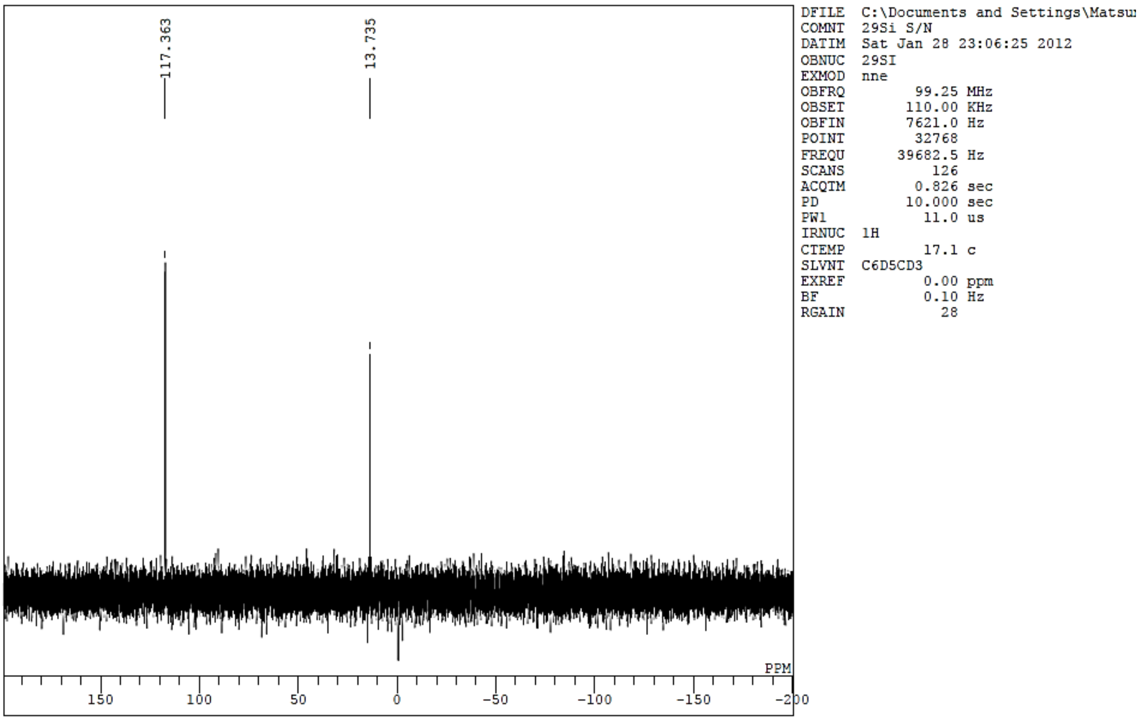

Supplementary Fig. 3 ^{29}Si NMR spectrum of 2 in toluene-d_8 at room temperature.

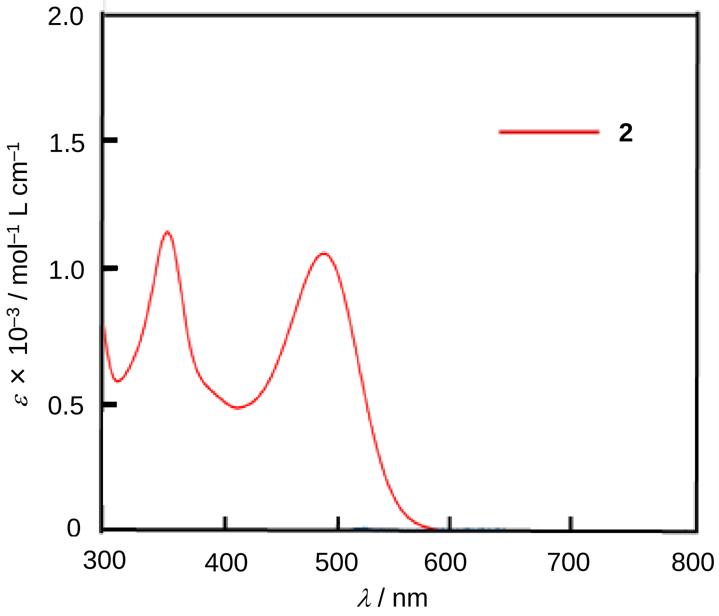

Supplementary Fig. 4 UV/Vis spectrum of 2 in benzene at room temperature.

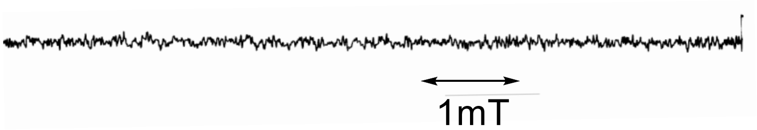

Supplementary Fig. 5 EPR spectrum of 2 in solid state at room temperature.

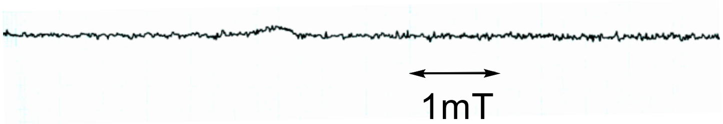

Supplementary Fig. 6 EPR spectrum of 2 in benzene at room temperature.

3. X-ray Crystallographic Analysis

Orange crystals of **2** were obtained from a benzene−THF (6:4) solution by slow evaporation under an argon atmosphere in a glovebox. A crystal specimen was mounted in a loop and used for data collection on a Rigaku R-AXIS IV^{++} imaging plate diffractometer using graphite-monochromated Mo Kα radiation. The data were corrected for Lorentz and polarization effects. An empirical absorption correction based on multi-scan was also applied. The structure was solved by a direct method using SHELXS-97[3]. Non-hydrogen atoms were refined anisotropically by the full-matrix least-squares method on F^2 for all reflections using SHELXL-2014/7[3,4]. All hydrogen atoms were generated by AFIX instructions and were not refined. All calculations were carried out using Yadokari-XG 2009[5].

Crystal data for **2** (123 K): $C_{24}H_{54}Si_4$, fw = 455.03, monoclinic, space group $C2/m$, a = 16.662(3), b = 11.5749(17), c = 8.5032(15) Å, β = 115.5534(9)°, V = 1479.5(4) Å^3, Z = 2, D_{calcd} = 1.021 g cm^{-3}, $R1$ = 0.053 (all data), wR_2 = 0.129 (all data), GOF = 1.11.

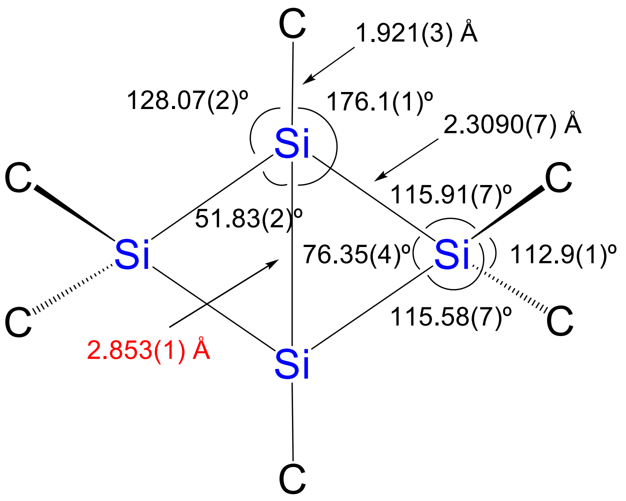

Supplementary Fig. 7 Structural parameters of 2.

4. Theoretical Calculations

All theoretical calculations were performed using Gaussian 09[6] on a Fujitsu PRIMERGY RX300 system of the Research Center for Computational Science, Japan. The structures were optimized at the B3LYP/6-31G(d) level, and the optimization was confirmed by frequency calculations. The results are summarized in Supplementary Tables 1 and 2. The natural bond orbital (NBO) analysis of **2** was carried out at the B3LYP/6-31+G(d,p) level using the X-ray structure. The result is summarized in Supplementary Data 1–3. The TD-DFT calculation of **2** was performed at the B3LYP/6-31G(d) level using the optimized structure. The result is summarized in Supplementary Data 4.

Supplementary Table 1 Atomic Coordinates of the Optimized Structures of 2 and 5–10.

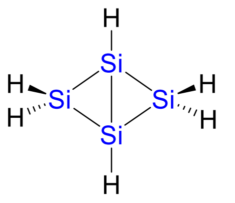

5$_{LB}$: long-bond isomer

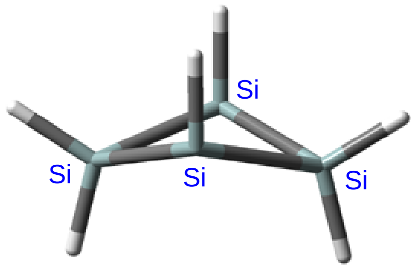

Atomic Type	Coordinates (Angstroms) X	Y	Z
Si	1.4301620	0.0000000	0.2799080
H	1.4883760	0.0000000	1.7803990
Si	0.0000000	1.7460420	-0.3171950
H	0.0000000	2.9844160	0.5047350
H	0.0000000	2.0815080	-1.7631240
Si	-1.4301620	0.0000000	0.2799080
H	-1.4883760	0.0000000	1.7803990
Si	0.0000000	-1.7460420	-0.3171950
H	0.0000000	-2.9844160	0.5047350
H	0.0000000	-2.0815080	-1.7631240

Framework group C_{2v}, energy: -1161.53798857 a.u.

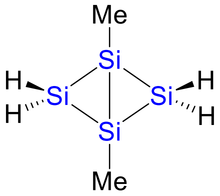

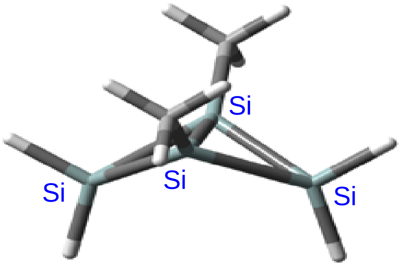

6$_{SB}$: short-bond isomer

Atomic		Coordinates (Angstroms)	
Type	X	Y	Z
Si	0.0000000	1.1917620	0.1456970
Si	-1.7257110	0.0000000	-0.8252850
H	-2.9650390	0.0000000	0.0032940
H	-2.1040610	0.0000000	-2.2685040
Si	0.0000000	-1.1917620	0.1456970
Si	1.7257110	0.0000000	-0.8252850
H	2.9650390	0.0000000	0.0032940
H	2.1040610	0.0000000	-2.2685040
C	0.0000000	2.7482490	1.2363330
H	0.8894340	2.7670260	1.8738320
H	0.0000000	3.6496120	0.6137800
H	-0.8894340	2.7670260	1.8738320
C	0.0000000	-2.7482490	1.2363330
H	0.0000000	-3.6496120	0.6137800
H	0.8894340	-2.7670260	1.8738320
H	-0.8894340	-2.7670260	1.8738320

Framework group C_{2v}, energy: -1240.17839296 a.u.

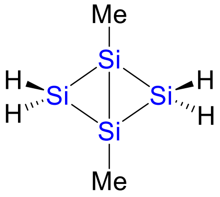

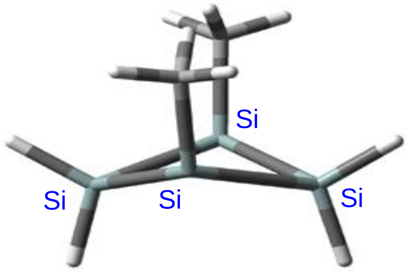

6$_{LB}$: long-bond isomer

Atomic		Coordinates (Angstroms)	
Type	X	Y	Z

Si	0.0000000	1.4560240	-0.2077120
Si	-1.7331430	0.0000000	-0.7847800
H	-2.9717740	0.0000000	0.0451230
H	-2.1010590	0.0000000	-2.2239130
Si	0.0000000	-1.4560240	-0.2077120
Si	1.7331430	0.0000000	-0.7847800
H	2.9717740	0.0000000	0.0451230
H	2.1010590	0.0000000	-2.2239130
C	0.0000000	-1.9104040	1.6602400
H	-0.8852580	-2.5138140	1.8903380
H	0.8852580	-2.5138140	1.8903380
H	0.0000000	-1.0439330	2.3315630
C	0.0000000	1.9104040	1.6602400
H	0.8852580	2.5138140	1.8903380
H	-0.8852580	2.5138140	1.8903380
H	0.0000000	1.0439330	2.3315630

Framework group C_{2v}, energy: -1240.17979576 a.u.

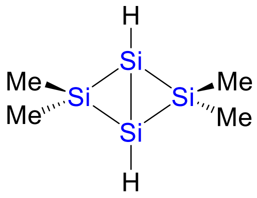

7

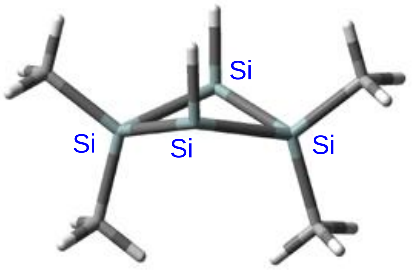

Atomic Type	Coordinates (Angstroms) X	Y	Z
Si	1.4093020	0.0000000	-0.4912060
H	1.5048110	0.0000000	-1.9939560
Si	0.0000000	1.7841520	0.0664380
Si	-1.4093020	0.0000000	-0.4912060
H	-1.5048110	0.0000000	-1.9939560
Si	0.0000000	-1.7841520	0.0664380
C	0.0000000	-2.2852760	1.8936310
H	0.8880160	-2.8832920	2.1307240
H	0.0000000	-1.4083230	2.5490600
H	-0.8880160	-2.8832920	2.1307240
C	0.0000000	-3.3072080	-1.0612440
H	0.8847810	-3.9265440	-0.8738910

H	-0.8847810	-3.9265440	-0.8738910
H	0.0000000	-3.0179140	-2.1163470
C	0.0000000	3.3072080	-1.0612440
H	0.8847810	3.9265440	-0.8738910
H	0.0000000	3.0179140	-2.1163470
H	-0.8847810	3.9265440	-0.8738910
C	0.0000000	2.2852760	1.8936310
H	0.8880160	2.8832920	2.1307240
H	-0.8880160	2.8832920	2.1307240
H	0.0000000	1.4083230	2.5490600

Framework group C_{2v}, energy: -1318.84627708 a.u.

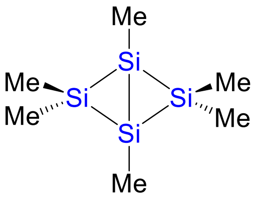

8

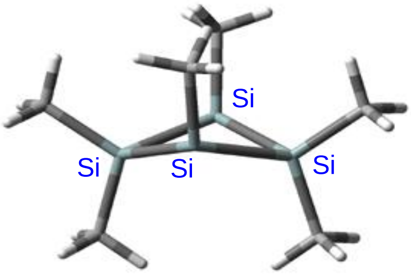

Atomic Type	Coordinates (Angstroms) X	Y	Z
Si	-0.0033530	1.4299610	-0.1252080
Si	1.7658120	0.0060760	0.4364990
Si	0.0033530	-1.4299610	-0.1252080
Si	-1.7658120	-0.0060760	0.4364990
C	0.0033530	1.8883660	-2.0000580
H	0.7333380	2.6867370	-2.1771970
H	0.2604080	1.0519320	-2.6604110
H	-0.9786920	2.2681960	-2.3029150
C	-0.0033530	-1.8883660	-2.0000580
H	0.9786920	-2.2681960	-2.3029150
H	-0.7333380	-2.6867370	-2.1771970
H	-0.2604080	-1.0519320	-2.6604110
C	-3.3090470	-0.0114770	-0.6730870
H	-3.9273840	0.8732190	-0.4804970
H	-3.0454900	-0.0171510	-1.7355530
H	-3.9243960	-0.8964040	-0.4720240
C	-2.2709350	-0.0022990	2.2644610
H	-2.8713780	0.8846480	2.4992740

H	-2.8660880	-0.8914920	2.5042460
H	-1.3934820	0.0021560	2.9190960
C	3.3090470	0.0114770	-0.6730870
H	3.9243960	0.8964040	-0.4720240
H	3.9273840	-0.8732190	-0.4804970
H	3.0454900	0.0171510	-1.7355530
C	2.2709350	0.0022990	2.2644610
H	2.8713780	-0.8846480	2.4992740
H	2.8660880	0.8914920	2.5042460
H	1.3934820	-0.0021560	2.9190960

Framework group C_2, energy: -1397.48494001 a.u.

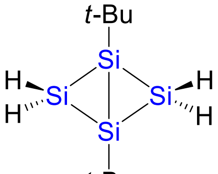

9

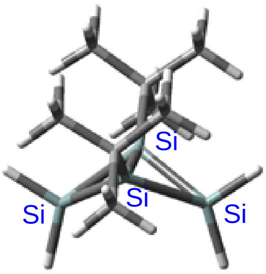

Atomic Type	Coordinates (Angstroms) X	Y	Z
Si	0.0198080	1.1916960	-0.4455880
Si	-1.6867390	0.0029920	-1.4618570
H	-2.9514710	0.0047810	-0.6724410
H	-2.0261470	0.0040660	-2.9150520
Si	-0.0198080	-1.1916960	-0.4455880
Si	1.6867390	-0.0029920	-1.4618570
H	2.9514710	-0.0047810	-0.6724410
H	2.0261470	-0.0040660	-2.9150520
C	-0.0109370	2.7792750	0.6620870
C	-0.0621350	4.0221800	-0.2506130
H	-0.9581880	4.0292680	-0.8818960
H	-0.0811200	4.9352160	0.3626360
H	0.8135760	4.0835330	-0.9071390
C	1.2606300	2.8218950	1.5316430
H	1.2567030	3.7280690	2.1549340
H	1.3237580	1.9577110	2.2028750
H	2.1719450	2.8433780	0.9231980
C	-1.2606300	2.7401910	1.5631670

H	-1.2478890	1.8760930	2.2372330
H	-1.3025720	3.6472280	2.1838950
H	-2.1864970	2.6968460	0.9782780
C	0.0109370	-2.7792750	0.6620870
C	0.0621350	-4.0221800	-0.2506130
H	0.0811200	-4.9352160	0.3626360
H	-0.8135760	-4.0835330	-0.9071390
H	0.9581880	-4.0292680	-0.8818960
C	-1.2606300	-2.8218950	1.5316430
H	-1.2567030	-3.7280690	2.1549340
H	-1.3237580	-1.9577110	2.2028750
H	-2.1719450	-2.8433780	0.9231980
C	1.2606300	-2.7401910	1.5631670
H	1.2478890	-1.8760930	2.2372330
H	1.3025720	-3.6472280	2.1838950
H	2.1864970	-2.6968460	0.9782780

Framework group C_2, energy: -1476.04907036 a.u.

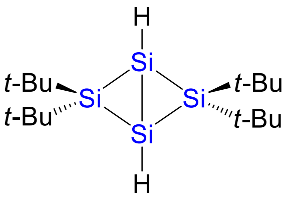

10

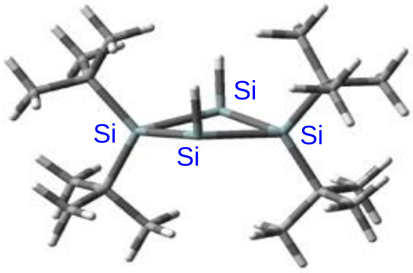

Atomic Type	Coordinates (Angstroms) X	Y	Z
Si	1.3901960	0.0109060	-0.2916490
H	1.6746580	-0.0229710	-1.7683720
Si	-0.0179950	1.8877550	-0.0223650
Si	-1.3901960	-0.0109060	-0.2916490
H	-1.6746580	0.0229710	-1.7683720
Si	0.0179950	-1.8877550	-0.0223650
C	0.0179950	2.7827160	1.7086980
C	-0.2134990	1.7664280	2.8405270
H	-0.2129380	2.2875470	3.8090770
H	-1.1757200	1.2520700	2.7406080
H	0.5775510	1.0122820	2.8757470
C	-1.0944750	3.8517920	1.7974520

H	-1.0840270	4.3102690	2.7969260
H	-0.9653120	4.6603720	1.0713730
H	-2.0901060	3.4175630	1.6476880
C	1.3947040	3.4461910	1.9469530
H	1.6276030	4.2214970	1.2116450
H	1.4067260	3.9229220	2.9381200
H	2.2043660	2.7079830	1.9259160
C	-0.1344650	3.0958070	-1.5684470
C	0.3162310	2.3833670	-2.8604970
H	-0.2573260	1.4720920	-3.0550460
H	0.1694340	3.0587940	-3.7158550
H	1.3763200	2.1128260	-2.8309500
C	0.7549180	4.3460590	-1.3904850
H	0.6909780	4.9710710	-2.2932180
H	0.4461840	4.9710120	-0.5468930
H	1.8101790	4.0840660	-1.2505520
C	-1.6042830	3.5440670	-1.7569940
H	-2.0036670	4.0697460	-0.8837070
H	-1.6719940	4.2327750	-2.6121840
H	-2.2614520	2.6934490	-1.9673860
C	-0.0179950	-2.7827160	1.7086980
C	-1.3947040	-3.4461910	1.9469530
H	-1.6276030	-4.2214970	1.2116450
H	-1.4067260	-3.9229220	2.9381200
H	-2.2043660	-2.7079830	1.9259160
C	0.2134990	-1.7664280	2.8405270
H	1.1757200	-1.2520700	2.7406080
H	-0.5775510	-1.0122820	2.8757470
H	0.2129380	-2.2875470	3.8090770
C	1.0944750	-3.8517920	1.7974520
H	0.9653120	-4.6603720	1.0713730
H	2.0901060	-3.4175630	1.6476880
H	1.0840270	-4.3102690	2.7969260
C	0.1344650	-3.0958070	-1.5684470
C	-0.3162310	-2.3833670	-2.8604970
H	-1.3763200	-2.1128260	-2.8309500
H	0.2573260	-1.4720920	-3.0550460
H	-0.1694340	-3.0587940	-3.7158550
C	-0.7549180	-4.3460590	-1.3904850
H	-0.6909780	-4.9710710	-2.2932180
H	-0.4461840	-4.9710120	-0.5468930
H	-1.8101790	-4.0840660	-1.2505520
C	1.6042830	-3.5440670	-1.7569940

H	2.0036670	-4.0697460	-0.8837070
H	1.6719940	-4.2327750	-2.6121840
H	2.2614520	-2.6934490	-1.9673860

Framework group C_2, energy: -1790.55853171 a.u.

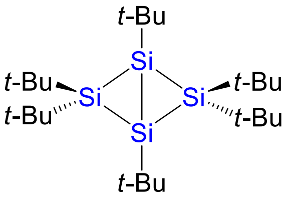

2

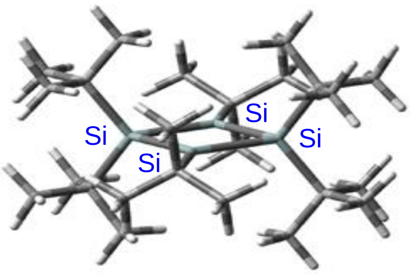

Atomic Type	Coordinates (Angstroms) X	Y	Z
Si	1.6970560	0.0000000	0.0000000
Si	0.0000000	0.0000000	1.6970560
Si	-1.6970560	0.0000000	0.0000000
Si	0.0000000	0.0000000	-1.6970560
C	3.5570560	0.0000000	0.0000000
C	4.0711190	1.4516680	0.0000000
C	4.0711190	-0.7258340	1.2571810
C	4.0711190	-0.7258340	-1.2571810
C	-3.5570560	0.0000000	0.0000000
C	-4.0711190	-1.4516680	0.0000000
C	-4.0711190	0.7258340	-1.2571810
C	-4.0711190	0.7258340	1.2571810
C	0.0000000	1.3152190	3.0122750
C	0.0000000	-1.3152190	3.0122750
C	0.0000000	1.3152190	-3.0122750
C	0.0000000	-1.3152190	-3.0122750
C	0.0000000	2.7232520	2.3406780
C	-1.2735080	1.1635290	3.9004000
C	1.2735080	1.1635290	3.9004000
C	0.0000000	-2.7232520	2.3406780
C	1.2735080	-1.1635290	3.9004000
C	-1.2735080	-1.1635290	3.9004000
C	0.0000000	2.7232520	-2.3406780
C	1.2735080	1.1635290	-3.9004000

C	-1.2735080	1.1635290	-3.9004000
C	0.0000000	-2.7232520	-2.3406780
C	-1.2735080	-1.1635290	-3.9004000
C	1.2735080	-1.1635290	-3.9004000
H	5.1711190	1.4516680	0.0000000
H	3.7049730	1.9708570	0.8979870
H	3.7049730	1.9708570	-0.8979870
H	5.1711190	-0.7258340	1.2571810
H	3.7049730	-1.7631080	1.2578190
H	3.7049730	-0.2077490	2.1558060
H	5.1711190	-0.7258340	-1.2571810
H	3.7049730	-0.2077490	-2.1558060
H	3.7049730	-1.7631080	-1.2578190
H	-5.1711190	-1.4516680	0.0000000
H	-3.7049730	-1.9708570	0.8979870
H	-3.7049730	-1.9708570	-0.8979870
H	-5.1711190	0.7258340	-1.2571810
H	-3.7049730	0.2077490	-2.1558060
H	-3.7049730	1.7631080	-1.2578190
H	-5.1711190	0.7258340	1.2571810
H	-3.7049730	1.7631080	1.2578190
H	-3.7049730	0.2077490	2.1558060
H	0.0000000	3.5010690	3.1184950
H	-0.8979870	2.8314700	1.7146510
H	0.8979870	2.8314700	1.7146510
H	-1.2735080	1.9413460	4.6782180
H	-1.2741460	0.1711620	4.3749600
H	-2.1721330	1.2709660	3.2751550
H	1.2735080	1.9413460	4.6782180
H	2.1721330	1.2709660	3.2751550
H	1.2741460	0.1711620	4.3749600
H	0.0000000	-3.5010690	3.1184950
H	0.8979870	-2.8314700	1.7146510
H	-0.8979870	-2.8314700	1.7146510
H	1.2735080	-1.9413460	4.6782180
H	1.2741460	-0.1711620	4.3749600
H	2.1721330	-1.2709660	3.2751550
H	-1.2735080	-1.9413460	4.6782180
H	-2.1721330	-1.2709660	3.2751550
H	-1.2741460	-0.1711620	4.3749600
H	0.0000000	3.5010690	-3.1184950
H	0.8979870	2.8314700	-1.7146510
H	-0.8979870	2.8314700	-1.7146510

H	1.2735080	1.9413460	-4.6782180
H	1.2741460	0.1711620	-4.3749600
H	2.1721330	1.2709660	-3.2751550
H	-1.2735080	1.9413460	-4.6782180
H	-2.1721330	1.2709660	-3.2751550
H	-1.2741460	0.1711620	-4.3749600
H	0.0000000	-3.5010690	-3.1184950
H	-0.8979870	-2.8314700	-1.7146510
H	0.8979870	-2.8314700	-1.7146510
H	-1.2735080	-1.9413460	-4.6782180
H	-1.2741460	-0.1711620	-4.3749600
H	-2.1721330	-1.2709660	-3.2751550
H	1.2735080	-1.9413460	-4.6782180
H	2.1721330	-1.2709660	-3.2751550
H	1.2741460	-0.1711620	-4.3749600

Framework group C_{2h}, energy: -2105.04161461 a.u.

Supplementary Table 2 Structural Parameters of 2 and 5–10.

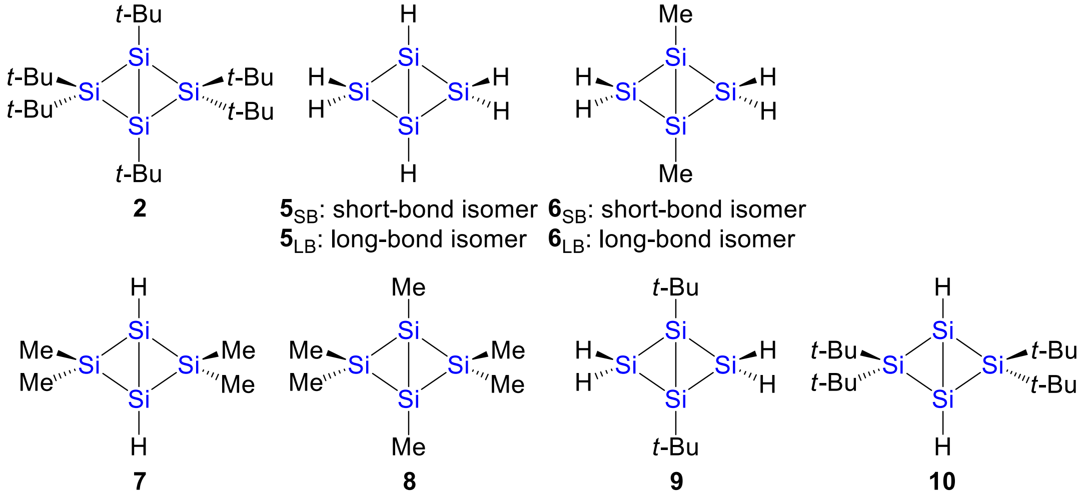

Compound	r / Å[a]	ϕ / °[a]	θ / °[a]	Reference
**5**$_{SB}$[a]	2.380	120.0	146.1	7
**5**$_{LB}$[a]	2.859	142.2	92.20	8
**6**$_{SB}$[a]	2.384	121.27	144.98	This work
**6**$_{LB}$[a]	2.912	143.17	103.67	This work
**7**	2.819	145.29	93.64	This work
**8**	2.860	144.71	103.74	This work
**9**	2.384	120.20	145.71	This work
**10**	2.781	163.76	100.84	This work
**2**	2.816	180.00	179.91	This work
**2** (X-ray)	2.853(1)	180.0	176.1(1)	This work

[a]The terms r, ϕ, and θ denote the following structural parameters. The terms SB and LB denote short-bond and long-bond isomers, respectively.

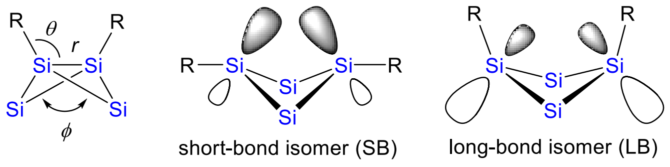

Supplementary References

1. Kyushin, S., Sakurai, H. & Matsumoto, H. Hepta-tert-butylcyclotetrasilane: a highly crowded cyclotetrasilane. *J. Organomet. Chem.* **499**, 235–240 (1995).
2. Kyushin, S., Kawai, H. & Matsumoto, H. (*trans*-1,2,2,3,4,4-Hexa-*tert*-butyl-1,3-cyclotetra-silanediyl)dipotassium: supramolecular structure of the silylpotassium–benzene complex. *Organometallics* **23**, 311–313 (2004).
3. Sheldrick, G. M. A short history of *SHELX*. *Acta Crystallogr., Sect. A* **64**, 112–122 (2008).
4. Sheldrick, G. M. Crystal structure refinement with *SHELXL*. *Acta Crystallogr., Sect. C* **71**, 3–8 (2015).
5. Kabuto, C., Akine, S., Nemoto, T. & Kwon, E. Release of software (Yadokari-XG 2009) for crystal structure analyses. *J. Cryst. Soc. Jpn.* **51**, 218– 224 (2009).
6. Frisch, M. J., Trucks, G. W., Schlegel, H. B., Scuseria, G. E., Robb, M. A., Cheeseman, J. R., Scalmani, G., Barone, V., Mennucci, B., Petersson, G. A., Nakatsuji, H., Caricato, M., Li, X., Hratchian, H. P., Izmaylov, A. F., Bloino, J., Zheng, G., Sonnenberg, J. L., Hada, M., Ehara, M., Toyota, K., Fukuda, R., Hasegawa, J., Ishida, M., Nakajima, T., Honda, Y., Kitao, O., Nakai, H., Vreven, T., Montgomery Jr., J. A., Peralta, J. E., Ogliaro, F., Bearpark, M., Heyd, J. J., Brothers, E., Kudin, K. N., Staroverov, V. N., Keith, T., Kobayashi, R., Normand, J., Raghavachari, K., Rendell, A., Burant, J. C., Iyengar, S. S., Tomasi, J., Cossi, M., Rega, N., Millam, J. M., Klene, M., Knox, J. E., Cross, J. B., Bakken, V., Adamo, C., Jaramillo, J., Gomperts, R., Stratmann, R. E., Yazyev, O., Austin, A. J., Cammi, R., Pomelli, C., Ochterski, J. W., Martin, R. L., Morokuma, K., Zakrzewski, V. G., Voth, G. A., Salvador, P., Dannenberg, J. J., Dapprich, S., Daniels, A. D., Farkas, O., Foresman, J. B., Ortiz, J. V., Cioslowski, J. & Fox, D. J. *Gaussian 09,* Revision E.01; Gaussian, Inc.: Wallingford, CT (2013).
7. Boatz, J. A. & Gordon, M. S. Bond-stretch isomerism in tetrasilabicyclo[1.1.0]butane derivatives. *Organometallics* **15**, 2118–2124 (1996).
8. Ueba-Ohshima, K., Iwamoto, T. & Kira, M. Synthesis, structure, and facile ring flipping of a bicyclo[1.1.0]tetrasilane. *Organometallics* **27**, 320–323 (2008).
