# Supplementary material for: Silicon–silicon π single bond
Source: Nat Commun. 2020 Aug 11;11:4009. doi: 10.1038/s41467-020-17815-z (PMC7419521; doi:10.1038/s41467-020-17815-z)
Supplement: Supplementary file 3 — Description of Additional Supplementary Information [file 41467_2020_17815_MOESM3_ESM.pdf]

Description of Additional Supplementary Files

File Name: Supplementary Data 1
Description: Wiberg bond index matrix of 2 in the NAO basis.

File Name: Supplementary Data 2
Description: Wiberg bond index, totals by atom of 2.

File Name: Supplementary Data 3
Description: Bond orbitals, coefficients and hybrids of 2.

File Name: Supplementary Data 4
Description: Transition energies, wavelengths and oscillator strengths of the transitions of 2.
